# Supplementary material for: Revealing the human mucinome
Source: Nat Commun. 2022 Jun 20;13:3542. doi: 10.1038/s41467-022-31062-4 (PMC9209528; doi:10.1038/s41467-022-31062-4)
Supplement: Supplementary file 1 — Supplementary Information [file 41467_2022_31062_MOESM1_ESM.pdf]

Supplementary Information for:

Revealing the human mucinome

Stacy A. Malaker[*‡], Nicholas M. Riley[‡], D. Judy Shon, Kayvon Pedram, Venkatesh Krishnan, Oliver Dorigo, Carolyn R. Bertozzi[*]

[‡] These authors contributed equally to the manuscript.

*Correspondence should be addressed to S.A.M. and C.R.B.
Email: stacy.malaker@yale.edu, bertozzi@stanford.edu

This PDF file includes:

- Supplementary Tables 1
- Supplementary Figures 1 to 9
- References

Uniprot	Protein	MucinScore	Previous Evidence?
O00592	Podocalyxin	2.48	yes
Q9Y5Y7	Lymphatic vessel endothelial hyaluronic acid receptor 1	5.33	no
Q4ZHG4	Fibronectin type III domain-containing protein 1	2.11	no*
Q6UX71	Plexin domain-containing protein 2	6.5	no*
P12259	Coagulation factor V	1.3	no*
Q76M96	Coiled-coil domain-containing protein 80	2.9	yes
P08174	Complement decay-accelerating factor (CD55)	5.11	yes
Q9NR99	Matrix-remodeling-associated protein 5	2.94	yes
Q7Z7G0	Target of Nesh-SH3	4.17	no
Q9Y279	V-set and immunoglobulin domain-containing protein 4	6	no
Q9HCU0	Endosialin	2.06	no
Q92954	Proteoglycan 4	12.2	yes
A1L4H1	Soluble scavenger receptor cysteine-rich domain-containing protein SSC5D	4	yes
Q9HBB8	Cadherin-related family member 5	3.75	no
P07359	Platelet glycoprotein Ib alpha chain	4.96	yes~
Q9BUN1	Protein MENT	2.42	no*
Q9BY67	Cell adhesion molecule 1	4	no
Q04756	Hepatocyte growth factor activator	1.88	no
P16070	CD44 antigen	2.32	yes
Q6EMK4	Vasorin	6	yes
Q9ULI3	Protein HEG homolog 1	1.77	yes
P12111	Collagen alpha-3(VI) chain	2.93	no
P25940	Collagen alpha-3(V) chain	3.15	no
O00468	Agrin	1.341	yes
Q9UGN4	CMRF35-like molecule 8	2.5	no
Q7Z7M0	Multiple epidermal growth factor-like domains protein 8	1.77	no

Supplementary Table 1. Overlapping mucin-domain glycoproteins from five ascites enrichments. The enriched mucins from five cancer patient ascites fluid samples were compared, and 26 proteins were found significantly enriched in all five samples. Uniprot numbers, protein names, MucinScores are listed for each overlapping mucin-domain glycoprotein. Proteins that were detected in SimpleCell data are listed as having previous evidence for a mucin domain, unless glycopeptides were found outside of the assigned mucin domain, indicated by an asterisk. A (~) denotes that this protein was not detected in the SimpleCell dataset, but is a known and/or canonical mucin-domain glycoprotein.

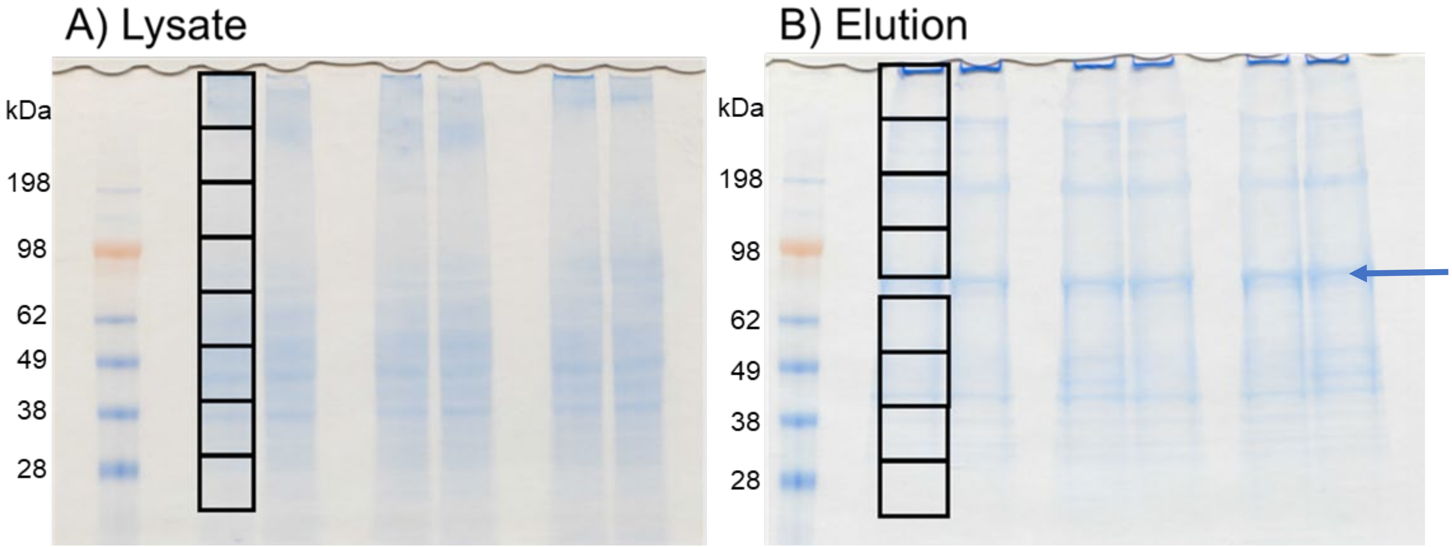

Supplementary Figure 1. Example slices cut from lysate and elution for in-gel digests. A) A total of 6 lanes were loaded with 6% of the enrichment input (~30 µg) and run on a 4-12% Bis-Tris gel for 1.5 h. Eight slices were cut from each lane; consecutive pairs of lanes were combined for a total of 3 replicates. B) A total of 6 lanes were loaded with the elution from 0.5 mg lysate in 100 µL beads (~200 µg StcE^{E447D}). Eight lanes were cut from each lane, avoiding the StcE^{E447D} band (arrow). Consecutive pairs of lanes were combined for a total of 3 replicates.

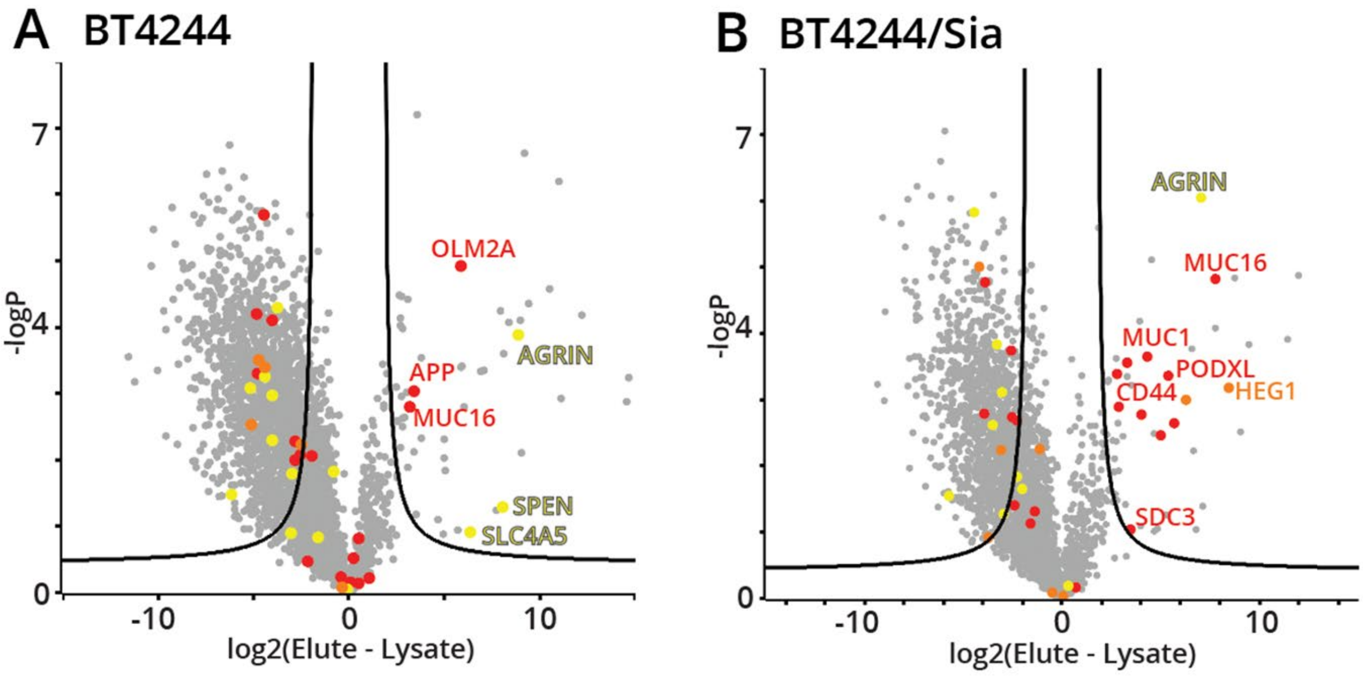

Supplementary Figure 2 BT4244^{E575A} enrichment of HeLa lysate. BT4244^{E575A} was conjugated to beads using reductive amination and HeLa lysate (A) or HeLa lysate pretreated with 100 nM VC sialidase overnight (B) was added to the beads. After binding, washing, and eluting, in-gel digest was performed on lysate alone and the elution of the enrichment. The samples were run on an Orbitrap Fusion Tribrid followed by a MaxQuant search. The data was processed using Perseus, and mucins were labeled according to the MucinScore. Red signified a score of >2 (high confidence), orange 2-1.5 (medium confidence), and yellow 1.5-1.2 (low confidence). Strongly enriched mucin-domain glycoproteins are labeled with their gene names. Significance testing was performed using a two-tailed t-test with 250 randomizations to correct for multiple comparisons, an FDR of 0.01, and an S0 value of 2.

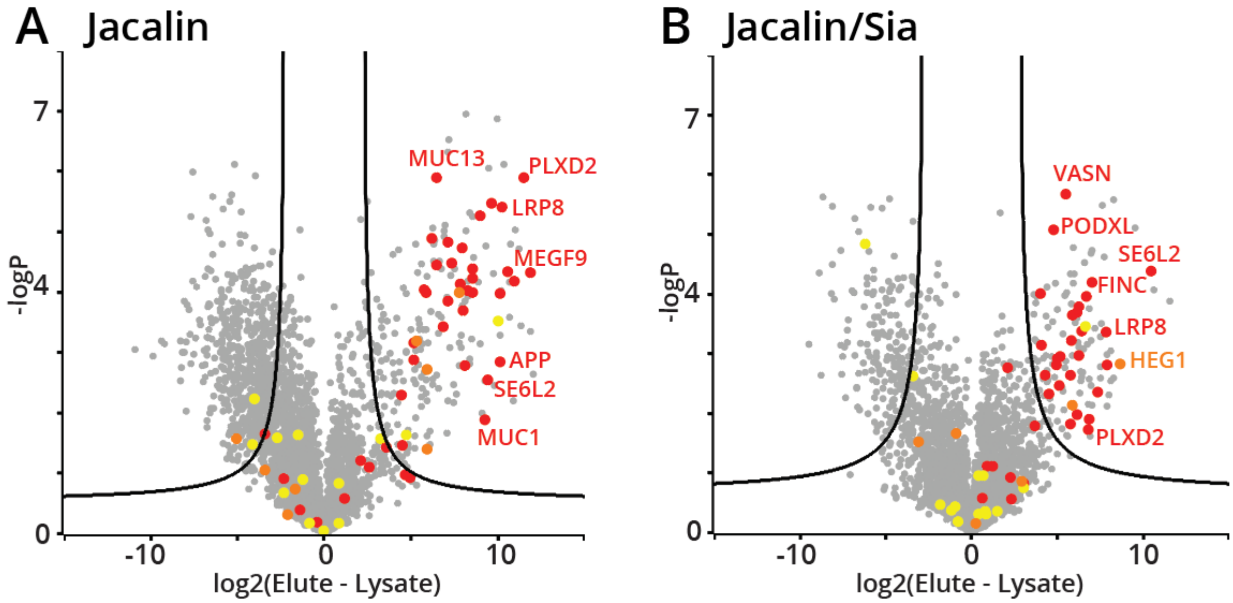

Supplementary Figure 3. Jacalin enrichment of HeLa lysate. Jacalin was conjugated to POROS-AL beads using reductive amidation and HeLa lysate (A) or HeLa lysate pretreated with 100 nM VC sialidase overnight (B) was added to the beads. After binding, washing, and eluting, in-gel digest was performed on lysate alone and the elution of the enrichment. The samples were run on an Orbitrap Fusion Tribrid followed by a MaxQuant search. The data was processed using Perseus, and mucins were labeled according to the MucinScore. Red signified a score of >2 (high confidence), orange 2-1.5 (medium confidence), and yellow 1.5-1.2 (low confidence). Strongly enriched proteins are labeled with their gene names. Significance testing was performed using a two-tailed t-test with 250 randomizations to correct for multiple comparisons, an FDR of 0.01, and an S0 value of 2.

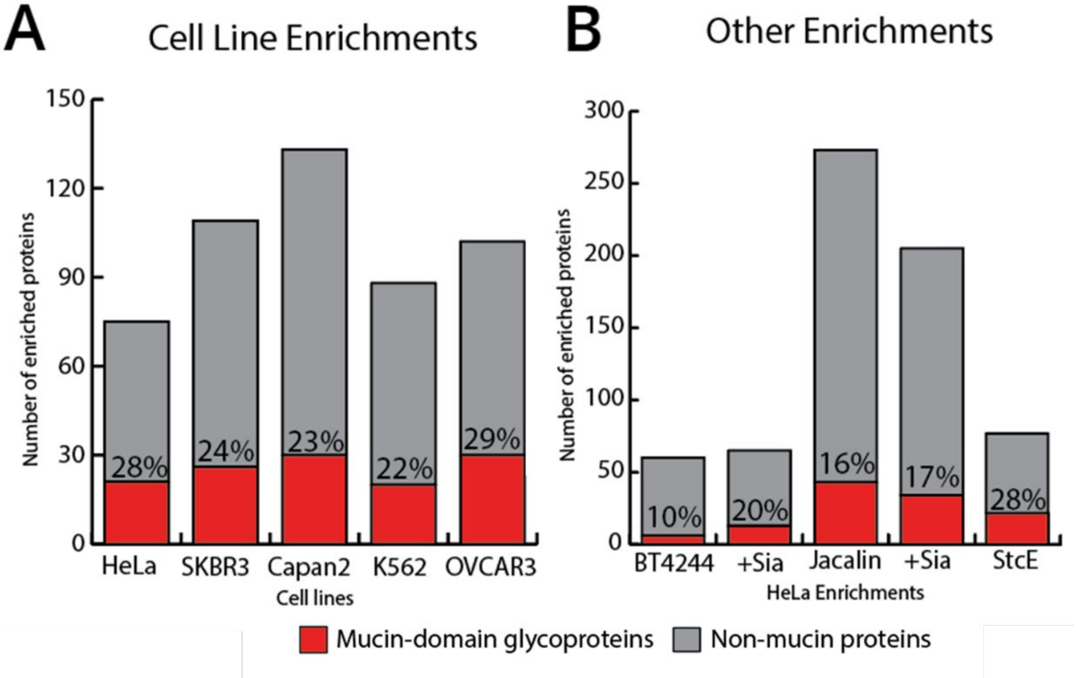

Supplementary Figure 4. Selectivity for mucin-domain glycoproteins in StcE^{E447D}, BT4244^{E575A}, and Jacalin enrichments. Statistically significantly enriched proteins were considered mucin-domain glycoproteins (red) if the MucinScore was higher than 1.2. All other proteins are considered non-mucin proteins (gray). The percentage of mucin-domain glycoproteins compared to total proteins is indicated on each bar. (A) **StcE^{E447D} cell line enrichments**. The average selectivity for mucin-domain glycoproteins was 25.1%. More information regarding enriched proteins can be found in Supplementary Data 4, including mucin-domain glycoprotein and non-mucin protein identifications, MucinScores, and GO terms. (B) BT4244^{E575A} and Jacalin enrichments. Given

BT4244's inability to accommodate sialic acid, the enrichment was 2x more selective for mucins after sialidase treatment. Jacalin exhibited poor selectivity for mucin-domain glycoproteins in both cases. The StcE^{E447D} enrichment of HeLa lysate is shown for ease of comparison.

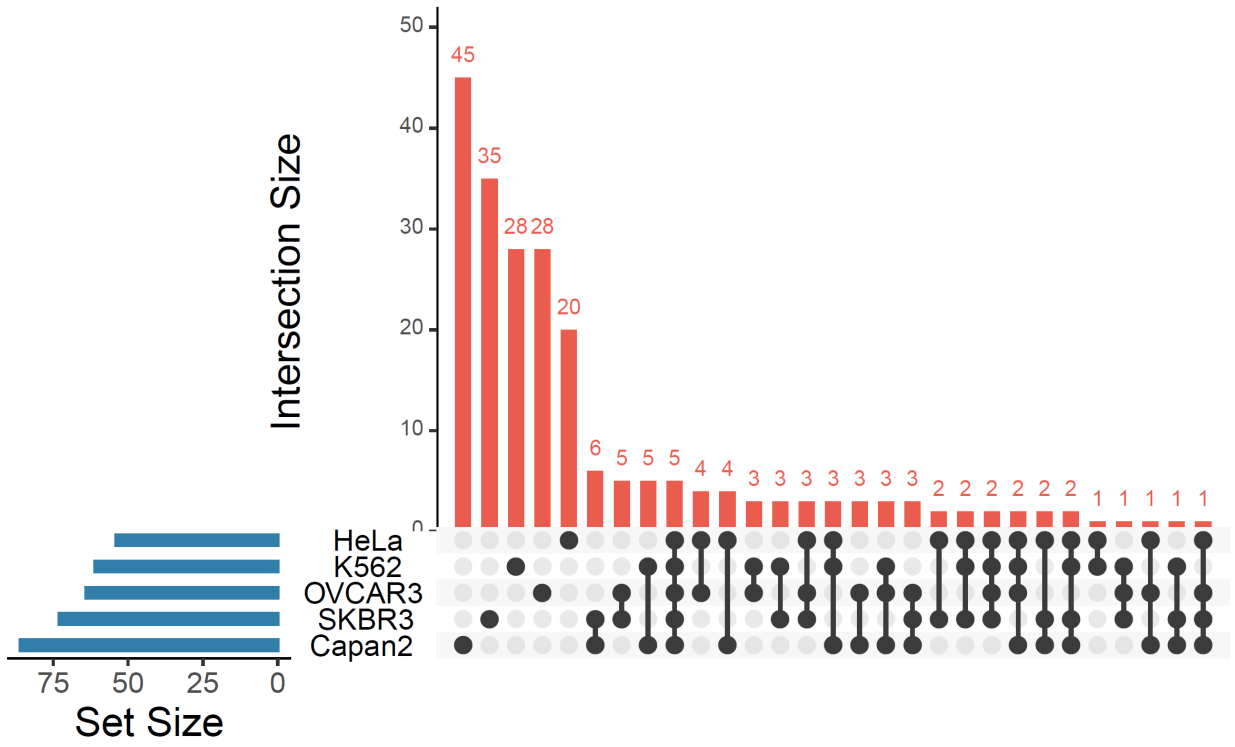

Supplementary Figure 5. Upset plot comparing enriched non-mucin proteins from five cell lysates. Figure was generated using Intervene UpSet tool. The majority of non-mucin proteins were found only in one cell line; however, 5 non-mucin proteins overlapped in all five lysates. Information about the overlapping non-mucin proteins can be found in Supplementary Data 4, including Uniprot IDs, protein names, and enriched GO terms.

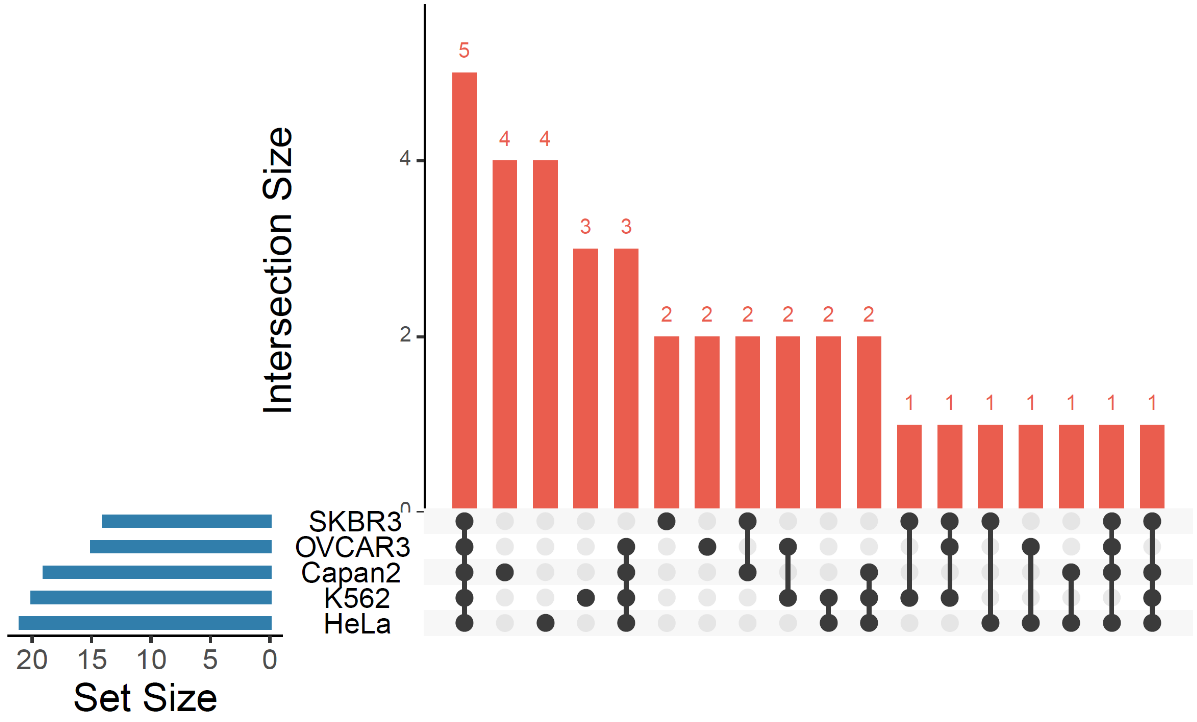

Supplementary Figure 6. Upset plot comparing unenriched mucin-domain glycoproteins from five cell lysates. Figure was generated using Intervene UpSet tool. Five mucin-domain glycoproteins were consistently

not enriched in the cell lysate samples. Information about these five proteins can be found in Supplementary Data 5, including Uniprot IDs, protein names, and enriched GO terms.

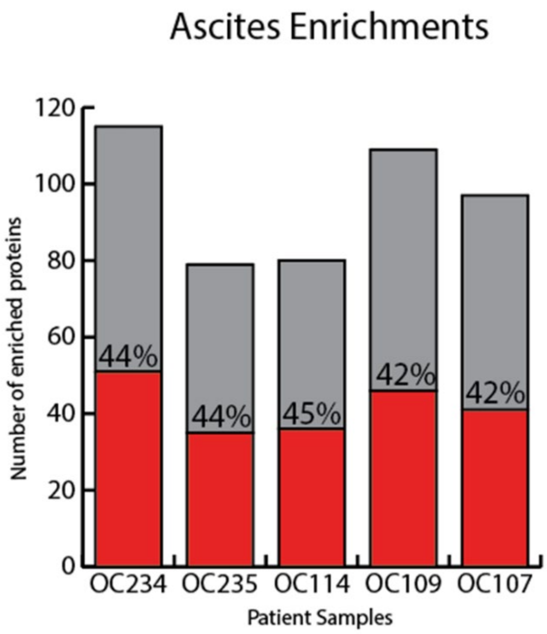

Supplementary Figure 7. Selectivity for mucin-domain glycoproteins in StcE^{E447D} ascites enrichments. . Statistically significantly enriched proteins were considered mucin-domain glycoproteins (red) if the MucinScore was higher than 1.2. All other proteins are considered non-mucin proteins (gray). The percentage of mucin-domain glycoproteins compared to total proteins is indicated on each bar. The average selectivity was 43.4%.

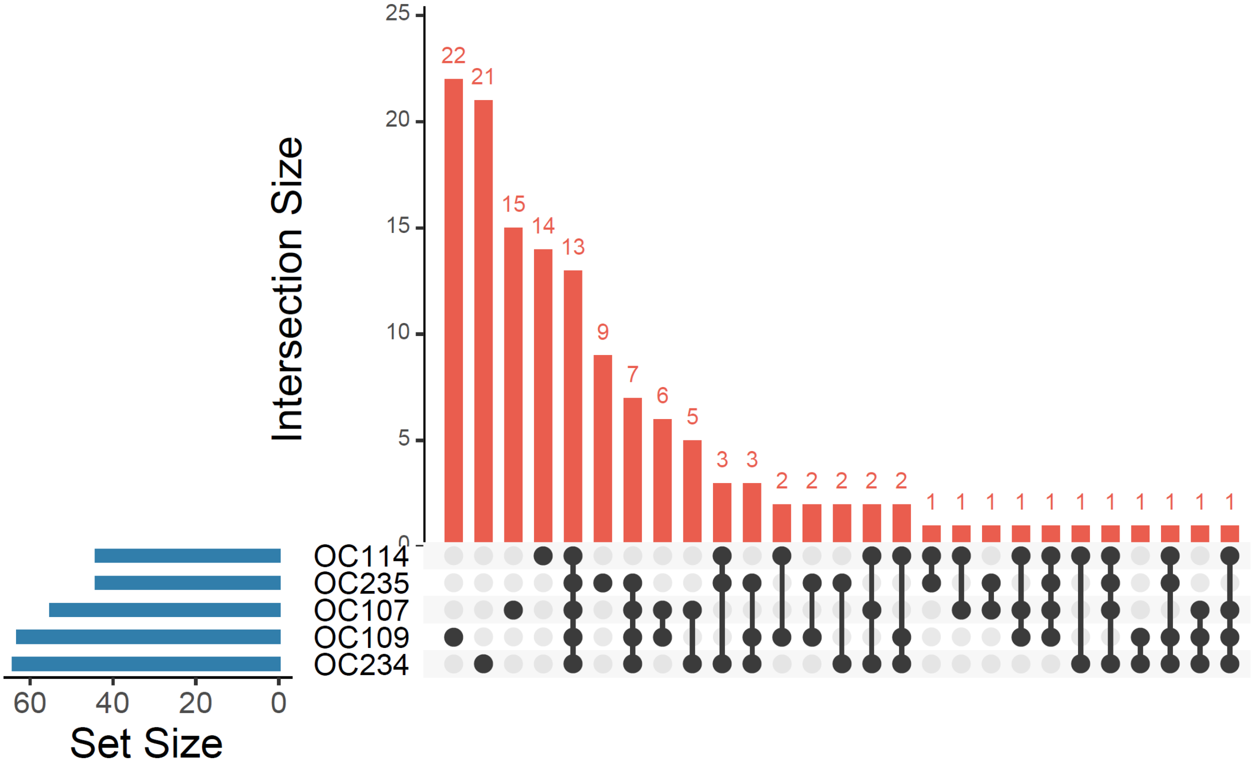

Supplementary Figure 8. Upset plot comparing enriched non-mucin proteins from five ascites patient samples. Figure was generated using Intervene UpSet tool. The majority of proteins are found only in one sample; however, 13 non-mucin proteins overlapped in all five patient samples. Information about the

overlapping non-mucin proteins can be found in Supplementary Data 7, including Uniprot IDs, protein names, and enriched GO terms.

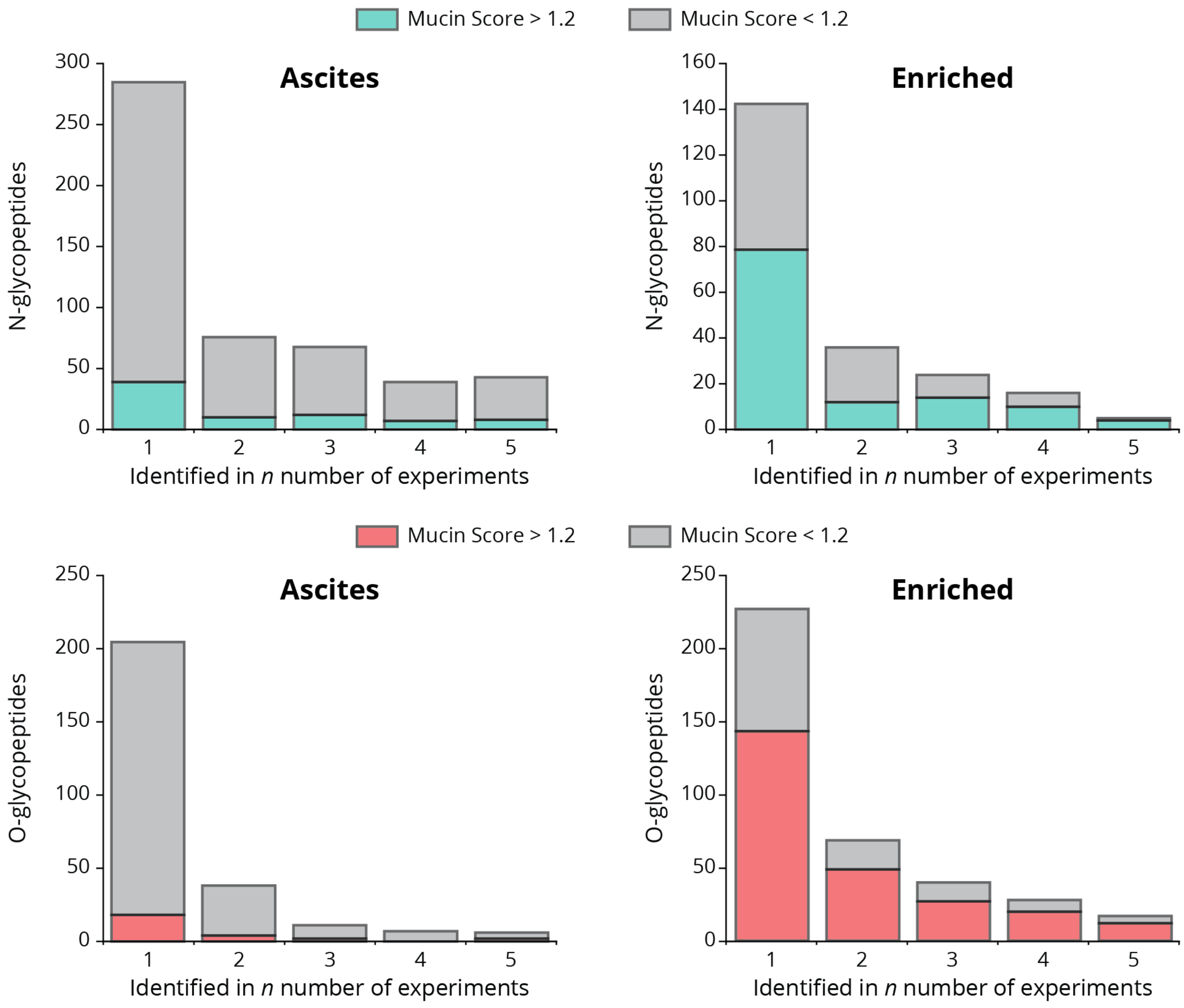

Supplementary Figure 9. Overlap in glycopeptides between ascites samples. Bar graphs show the number of N-glycopeptides (top, green) and O-glycopeptides (bottom, red) that were detected in *n* number of patient samples either in the unenriched ascites fluid (ascites) or the mucinome enriched samples (enriched).

Supplementary References

1. Steentoft, C. *et al.* Precision mapping of the human O-GalNAc glycoproteome through SimpleCell technology. *EMBO J.* **32**, 1478–1488 (2013).
