# Supplementary material for: Revealing the human mucinome
Source: Nat Commun. 2022 Jun 20;13:3542. doi: 10.1038/s41467-022-31062-4 (PMC9209528; doi:10.1038/s41467-022-31062-4)
Supplement: Supplementary file 3 — Description of Additional Supplementary Files [file 41467_2022_31062_MOESM3_ESM.pdf]

Description of Additional Supplementary Files for:

Revealing the human mucinome

Stacy A. Malaker[*‡], Nicholas M. Riley[‡], D. Judy Shon, Kayvon Pedram, Venkatesh Krishnan, Oliver Dorigo, Carolyn R. Bertozzi[*]

[‡] These authors contributed equally to the manuscript.

*Correspondence should be addressed to S.A.M. and C.R.B.
Email: stacy.malaker@yale.edu, bertozzi@stanford.edu

File Name: Supplementary Data 1
Description: Mucin Domain Candidacy Output
Legend: This file provides a summary of the mucin domain information calculated by the mucin domain candidacy algorithm for 20,191 proteins in the human proteome.

File Name: Supplementary Data 2
Description: Mucin Domain Locations
Legend: This file shows which predicted O-glycosites comprise mucin domains annotated by the mucin domain candidacy algorithm.

File Name: Supplementary Data 3
Description: Cell Line Perseus Results
Legend: Raw data from cell line controls and enrichments were searched using MaxQuant version 1.6.3.4 as described in the Methods section. A Boolean value "IsAMucin" was also appended to each protein, with the value set as true if the Mucin Score was greater than 1. Mucin Scores and IsAMucin were input manually into MQ 'protein groups' txt files for manipulation in Perseus. Significance testing was performed in Perseus using a two-tailed t-test with 250 randomizations to correct for multiple comparisons, an FDR of 0.01, and an S0 value of 2 (all volcano plots), or in Microsoft Excel using a two-tailed t-test with heteroscedastic variance. After generating these data, we collated all of the results into this excel file.

File Name: Supplementary Data 4
Description: New vs Known Mucin Lists
Legend: Comparison of SimpleCell dataset to our list of enriched proteins assigned as mucin-domain glycoproteins. To consider a mucin-domain glycoprotein as previously unknown, more than 1 glycopeptide had to be detected from within the assigned mucin domain. Additionally, if the protein was a canonical (e.g. MUC15) or confirmed (e.g. Gp1bα) mucin-domain glycoprotein, these were considered as previously described/known proteins.

File Name: Supplementary Data 5
Description: Cell Line_Enriched Mucins vs NonMucins
Legend: All statistically significantly enriched proteins (listed as Uniprot numbers) from cell lysate enrichments were separated by Mucin Domain Score into "Mucin" and "NonMucin" tabs with a cutoff value of 1.2. "Master_Mucin" and "Master_NonMucin" tabs were generated to collate lists of each from the five cell lines. Information about highly overlapping proteins from each group are listed in the master tabs.

File Name: Supplementary Data 6
Description: Cell Line_Unenriched Mucins
Legend: All proteins assigned as mucin-domain glycoproteins that did not fall into the category of statistically significantly enriched were collected into individual tabs. Uniprot IDs from each cell lysate was collected into "Master_Unenriched Mucins" and used to identify mucin-domain glycoproteins that were consistently not enriched with our procedure.

File Name: Supplementary Data 7
Description: Ascites Perseus Results
Legend: Raw data from ascites controls and enrichments were searched using MaxQuant version 1.6.3.4 as described in the Methods section. A Boolean value “IsAMucin” was also appended to each protein, with the value set as true if the Mucin Score was greater than 1. Mucin Scores and IsAMucin were input manually into MQ ‘protein groups’ txt files for manipulation in Perseus. Significance testing was performed in Perseus using a two-tailed t-test with 250 randomizations to correct for multiple comparisons, an FDR of 0.01, and an S0 value of 2 (all volcano plots), or in Microsoft Excel using a two-tailed t-test with heteroscedastic variance. After generating these data, we collated all of the results into this excel file.

File Name: Supplementary Data 8
Description: Ascites_Enriched Mucins vs NonMucins
Legend: All statistically significantly enriched proteins (listed as Uniprot numbers) from ascites enrichments were separated by Mucin Domain Score into “Mucin” and “NonMucin” tabs with a cutoff value of 1.2. “Master_Mucin” and “Master_NonMucin” tabs were generated to collate lists of each from the five cell lines. Information about highly overlapping proteins from each group are listed in the master tabs.

File Name: Supplementary Data 9
Description: Glycan Databases Used.
Legend: This file provides the glycan compositions used for N- and O-glycopeptide searches.

File Name: Supplementary Data 10
Description: Nglycopeptides Ascites and Elute
Legend: Ascites control and enrichment files were loaded into MetaMorpheus O-Pair Search in groups of 8 and a “Glyco” search task was selected. O-glycopeptides were searched with the following parameters: O-glycan database “Oglycan.gdb” (the default 12-glycan database), keep top 50 candidates, Dissociation type “HCD” and child scan “null”, 4 maximum Oglycan allowed, with OxoniumIonFit on. Data from each replicate can be found in individual tabs.

File Name: Supplementary Data 11
Description: Oglycopeptides Ascites and Elute
Legend: Ascites control and enrichment files were loaded into MetaMorpheus O-Pair Search in groups of 8 and a “Glyco” search task was selected. N-glycopeptides were searched with the following parameters: “NGlycan182.gdb” database, keep top 50 candidates, Dissociation type “HCD” and child scan “null”, with OxoniumIonFit on. Data from each replicate can be found in individual tabs.

File Name: Supplementary Data 12
Description: Nglycopeptide-Glycan Network Data.
Legend: This file provides the necessary data to recreate the glycopeptide-glycan network in Figure 6D, which was created in R 3.5.1 using the igraph library[75].

File Name: Supplementary Data 13
Description: Oglycopeptide-Glycan Network Data.
Legend: This file provides the necessary data to recreate the glycopeptide-glycan network in Figure 6E, which was created in R 3.5.1 using the igraph library[75].

Supplementary Software 1
Description: Mucin Candidacy Algorithm.
Legend: These files can be copied and run through any integrated development environment that can run C# code, e.g., Visual Studio. Instructions for running this code are provided in the readme.txt file and necessary input files from NetOGlyc output of the human proteome are provided.
