# Supplementary material for: Revealing the human mucinome
Source: Nat Commun. 2022 Jun 20;13:3542. doi: 10.1038/s41467-022-31062-4 (PMC9209528; doi:10.1038/s41467-022-31062-4)
Supplement: Supplementary file 18 — Reporting Summary [file 41467_2022_31062_MOESM18_ESM.pdf]

nature research

Corresponding author(s): Carolyn R. Bertozzi, Stacy A. Malaker

Last updated by author(s): May 2, 2022

Reporting Summary

Nature Research wishes to improve the reproducibility of the work that we publish. This form provides structure for consistency and transparency in reporting. For further information on Nature Research policies, see our Editorial Policies and the Editorial Policy Checklist.

Statistics

For all statistical analyses, confirm that the following items are present in the figure legend, table legend, main text, or Methods section.

n/a	Confirmed	
☐	☒	The exact sample size (*n*) for each experimental group/condition, given as a discrete number and unit of measurement
☐	☒	A statement on whether measurements were taken from distinct samples or whether the same sample was measured repeatedly
☐	☒	The statistical test(s) used AND whether they are one- or two-sided<br>*Only common tests should be described solely by name; describe more complex techniques in the Methods section.*
☒	☐	A description of all covariates tested
☒	☐	A description of any assumptions or corrections, such as tests of normality and adjustment for multiple comparisons
☐	☒	A full description of the statistical parameters including central tendency (e.g. means) or other basic estimates (e.g. regression coefficient) AND variation (e.g. standard deviation) or associated estimates of uncertainty (e.g. confidence intervals)
☐	☒	For null hypothesis testing, the test statistic (e.g. *F*, *t*, *r*) with confidence intervals, effect sizes, degrees of freedom and *P* value noted<br>*Give P values as exact values whenever suitable.*
☒	☐	For Bayesian analysis, information on the choice of priors and Markov chain Monte Carlo settings
☒	☐	For hierarchical and complex designs, identification of the appropriate level for tests and full reporting of outcomes
☒	☐	Estimates of effect sizes (e.g. Cohen's *d*, Pearson's *r*), indicating how they were calculated

Our web collection on statistics for biologists contains articles on many of the points above.

Software and code

Policy information about availability of computer code

Data collection	Data was collected on a Thermo Orbitrap Fusion Tribrid using Xcalibur software (v4.1).
Data analysis	MucinScores were determined using a Mucin Candidacy Algorithm that compiled data from several sources, including NetOGlyc4.0 for O-glycosite prediction, PhosphoSitePlus (v6.6.04) for known phosphosite information, and Uniprot for cellular localization and known phosphosite information. This Mucin Candidacy Algorithm is not designed to be run by users routinely but instead provides a one-time scoring output on the entire human proteome, which can then be mapped to (glyco)proteomics results. This output has been provided, and both input files and the source code to generate the output have also been provided. Proteomic data was analyzed using MaxQuant (v1.6.10.43) and processed using Perseus and Microsoft Excel. Glycoproteomic data was searched with the OPair Search node of Meta Morpheus (v0.0.308) and analyzed using Microsoft Excel, OriginPro 2022, and R v3.5.1.

For manuscripts utilizing custom algorithms or software that are central to the research but not yet described in published literature, software must be made available to editors and reviewers. We strongly encourage code deposition in a community repository (e.g. GitHub). See the Nature Research guidelines for submitting code & software for further information.

Data

Policy information about availability of data

All manuscripts must include a data availability statement. This statement should provide the following information, where applicable:

- Accession codes, unique identifiers, or web links for publicly available datasets
- A list of figures that have associated raw data
- A description of any restrictions on data availability

The raw mass spectrometry data generated in this study have been deposited in the PRIDE database68 under accession code PXD024995 https://www.ebi.ac.uk/pride/archive/projects/PXD024995. The SimpleCell dataset from Clausen and colleagues was obtained from Steentoft et al. [51] Supplemental Table 2. The

proteomics data generated from the mucinome enrichments of cell lysates and ascites fluid, the outputs from the mucin candidacy algorithm, the glycan databases used for glycopeptide searches, the glycoproteomics data generated from the mucinome enrichments of ascites fluid, data to make the N- and O-glycopeptide networks, and data to recreate figures are provided in the Supplementary Information files as indicated in the text and below. Source data are provided with this paper.
Supplementary Data 1_Mucin Domain Candidacy Output. This file provides a summary of the mucin domain information calculated by the mucin domain candidacy algorithm for 20,191 proteins in the human proteome.
Supplementary Data 2_Mucin Domain Locations. This file shows which predicted O-glycosites comprised mucin domains annotated by the mucin domain candidacy algorithm.
Supplementary Data 3_Cell Line Perseus Results. This file provides data to recreate volcano plots for each cell line to show enriched mucin domain glycoproteins.
Supplementary Data 4_New vs Known Mucin Lists. This file details mucin-domain glycoprotein candidates found in the cell line and patient ascites fluid samples and indicates whether they have been documented as mucins before according to Steentoft et al. [51]
Supplementary Data 5_Cell Line_Enriched Mucins vs NonMucins. This file shows the mucins that were found in multiple cell lines and also shows which non mucins were identified.
Supplementary Data 6_Cell Line_Unenriched Mucins. This file provides the list of potential mucin candidates identified but were not enriched in cell lines.
Supplementary Data 7_Ascites Perseus Results. This file provides data to recreate volcano plots for each ascites fluid sample to show enriched mucin domain glycoproteins.
Supplementary Data 8_Ascites_Enriched Mucins vs NonMucins. This file shows the mucins that were found in multiple ascites fluid samples and also shows which non mucins were identified.
Supplementary Data 9_Glycan Databases Used. This file provides the glycan compositions used for N- and O-glycopeptide searches.
Supplementary Data 10_Nglycopeptides Ascites and Elute. This file provides N-glycopeptides identified (and relevant information about identifications) in the elution and non-enriched fractions of ascites fluid samples.
Supplementary Data 11_Oglycopeptides Ascites and Elute. This file provides O-glycopeptides identified (and relevant information about identifications) in the elution and non-enriched fractions of ascites fluid samples.
Supplementary Data 12_Nglycopeptide-Glycan Network Data. This file provides the necessary data to recreate the glycopeptide-glycan network in Figure 6D.
Supplementary Data 13_Oglycopeptide-Glycan Network Data. This file provides the necessary data to recreate the glycopeptide-glycan network in Figure 6E

Code Availability
Code for the mucinome candidacy algorithm is available as Supplementary Software 1.

Field-specific reporting

Please select the one below that is the best fit for your research. If you are not sure, read the appropriate sections before making your selection.

- [x] Life sciences
- [ ] Behavioural & social sciences
- [ ] Ecological, evolutionary & environmental sciences

For a reference copy of the document with all sections, see nature.com/documents/nr-reporting-summary-flat.pdf

Life sciences study design

All studies must disclose on these points even when the disclosure is negative.

Sample size	In this study, we used 5 cell lines and 5 ovarian cancer patient ascites fluid samples. All analyses were performed in triplicate to provide the statistical power needed for significance testing.
Data exclusions	No data were excluded from this study
Replication	All data collection was performed in triplicate; all attempts at data replication were successful
Randomization	This is not relevant to our study because cell lines were biological replicates and patient samples were not allocated into experimental groups.
Blinding	Not applicable because patient samples were not allocated into experimental groups.

Reporting for specific materials, systems and methods

We require information from authors about some types of materials, experimental systems and methods used in many studies. Here, indicate whether each material, system or method listed is relevant to your study. If you are not sure if a list item applies to your research, read the appropriate section before selecting a response.

Materials & experimental systems

n/a	Involved in the study	
☐	☒	Antibodies
☐	☒	Eukaryotic cell lines
☒	☐	Palaeontology and archaeology
☒	☐	Animals and other organisms
☐	☒	Human research participants
☒	☐	Clinical data
☒	☐	Dual use research of concern

Methods

n/a	Involved in the study	
☒	☐	ChIP-seq
☒	☐	Flow cytometry
☒	☐	MRI-based neuroimaging

Antibodies

Antibodies used	Abcam anti-MUC16 ab1107, LiCor IRDye® 800CW Goat anti-Mouse IgG 926-32210
Validation	An antibody was considered validated if it produced a band of the expected molecular weight for the target protein, MUC16. IgG was used as a secondary antibody for visualization, thus the same validation technique was used.

Eukaryotic cell lines

Policy information about cell lines

Cell line source(s)	HeLa: ATCC CCL-2, Capan-2: ATCC HTB-80, K562: ATCC CRL-3344, SKBR3: ATCC HTB-30, OVCAR3: ATCC HTB-161
Authentication	Not further authenticated
Mycoplasma contamination	All cell lines were tested weekly for Mycoplasma contamination and remained negative.
Commonly misidentified lines (See ICLAC register)	None of the cell lines used in this study were identified by the ICLAC register

Human research participants

Policy information about studies involving human research participants

Population characteristics	Ascites fluid from 5 female patients with gynecologic malignancies. All samples were de-identified prior to enrichment.
Recruitment	All of the samples were collected with informed consent under the approved IRB protocol
Ethics oversight	The IRB protocol was approved by the Dept. of Obstetrics and Gynecology, Stanford Hospital. The study design and conduct complied with all relevant regulations regarding the use of human study participants and was in accordance with the criteria set by the Declaration of Helsinki.

Note that full information on the approval of the study protocol must also be provided in the manuscript.
